# Supplementary material for: Predictable properties of fitness landscapes induced by adaptational tradeoffs
Source: eLife. 2020 May 19;9:e55155. doi: 10.7554/eLife.55155 (PMC7297540; doi:10.7554/eLife.55155)
Supplement: Transparent reporting form [file elife-55155-transrepform.docx]

***eLife’s* transparent reporting form**

We encourage authors to provide detailed information *within their submission* to facilitate the interpretation and replication of experiments. Authors can upload supporting documentation to indicate the use of appropriate reporting guidelines for health-related research (see [EQUATOR Network](http://www.equator-network.org/%20\t%20_blank)), life science research (see the [BioSharing Information Resource](https://biosharing.org/)), or the [ARRIVE guidelines](http://www.plosbiology.org/article/info:doi/10.1371/journal.pbio.1000412) for reporting work involving animal research. Where applicable, authors should refer to any relevant reporting standards documents in this form.

If you have any questions, please consult our Journal Policies and/or contact us: [editorial@elifesciences.org](mailto:editorial@elifesciences.org).

**Sample-size estimation**

- You should state whether an appropriate sample size was computed when the study was being designed
- You should state the statistical method of sample size computation and any required assumptions
- If no explicit power analysis was used, you should describe how you decided what sample (replicate) size (number) to use

Please outline where this information can be found within the submission (e.g., sections or figure legends), or explain why this information doesn’t apply to your submission:

No sample-size estimation was performed prior to the experiment. This was deemed unnecessary because (i) replicate experiments yield very similar results (growth curves), (ii) no hypothesis testing has been carried out based on the data, (iii) experimental data presented serve only as a qualitative demonstration of the assumptions of the mathematical model.

The number of replicate experiments is mentioned in Methods, section Dose-response curves. Data analysis and error estimation was based upon the assumed statistical independence of samples.

**Replicates**

- The data obtained should be provided and sufficient information should be provided to indicate the number of independent biological and/or technical replicates
- If you encountered any outliers, you should describe how these were handled
- Criteria for exclusion/inclusion of data should be clearly stated
- High-throughput sequence data should be uploaded before submission, with a private link for reviewers provided (these are available from both GEO and ArrayExpress)

Please outline where this information can be found within the submission (e.g., sections or figure legends), or explain why this information doesn’t apply to your submission:

Experimental design, including the number of replicates, is described in Methods, section Dose-response curves.

All replicates are biological replicates (bacterial cultures seeded independently from each other).

We only excluded growth curves that were clearly affected by experimental error (e.g., wells not fully sealed-off by transparent seal); their shape was dramatically different to the typical shape. The number of excluded replicates was less than 10% of all replicates.

- Raw data should be presented in figures whenever informative to do so (typically when N per group is less than 10)
- For each experiment, you should identify the statistical tests used, exact values of N, definitions of center, methods of multiple test correction, and dispersion and precision measures (e.g., mean, median, SD, SEM, confidence intervals; and, for the major substantive results, a measure of effect size (e.g., Pearson's r, Cohen's d)
- Report exact p-values wherever possible alongside the summary statistics and 95% confidence intervals. These should be reported for all key questions and not only when the p-value is less than 0.05.

Please outline where this information can be found within the submission (e.g., sections or figure legends), or explain why this information doesn’t apply to your submission:

Raw data (growth curves) were analysed using a non-standard method, therefore most of the information provided above does not apply. We nevertheless provide mean and standard errors of all measurements as determined by the algorithm described in Methods, section Dose-response curves, and reference Ojkic et al., 2019.

A statistical analysis was required only for Table 1 in the main article to detect the presence or absence of epistasis. The details can be found in the paragraph above the table.

(For large datasets, or papers with a very large number of statistical tests, you may upload a single table file with tests, Ns, etc., with reference to sections in the manuscript.)

**Group allocation**

- Indicate how samples were allocated into experimental groups (in the case of clinical studies, please specify allocation to treatment method); if randomization was used, please also state if restricted randomization was applied
- Indicate if masking was used during group allocation, data collection and/or data analysis

Please outline where this information can be found within the submission (e.g., sections or figure legends), or explain why this information doesn’t apply to your submission:

Groups were defined by mutant genotype and antibiotic concentration prior to experiment.

**Additional data files (“source data”)**

- We encourage you to upload relevant additional data files, such as numerical data that are represented as a graph in a figure, or as a summary table
- Where provided, these should be in the most useful format, and they can be uploaded as “Source data” files linked to a main figure or table
- Include model definition files including the full list of parameters used
- Include code used for data analysis (e.g., R, MatLab)
- Avoid stating that data files are “available upon request”

Please indicate the figures or tables for which source data files have been provided:

Data have been provided for Fig. 2A.
